# Bisulfite‐free *PCDHGB7* methylation in urine enables early noninvasive detection of urothelial carcinoma

**DOI:** 10.1002/btm2.70004

**Published:** 2025-02-26

**Authors:** Zhicong Yang, Qing Chen, Shihua Dong, Peng Xu, Zhanrui Mao, Yaping Dong, Wei Li, Wenxuan Li, Yang Han, Lihe Dai, Gehong Dong, Yong Zhang, Yinshan Li, Liang Cheng, Weimin Ci, Wenqiang Yu, Chuanliang Xu

**Affiliations:** ^1^ Shanghai Public Health Clinical Center, Huashan Hospital, Cancer Metastasis Institute, Institutes of Biomedical Sciences Fudan University Shanghai China; ^2^ Department of Urology, Changhai Hospital Naval Medical University Shanghai China; ^3^ Department of research and development Shanghai Epiprobe Biotechnology Co., Ltd Shanghai China; ^4^ Department of Urology, Shanghai General Hospital Shanghai Jiao Tong University School of Medicine Shanghai China; ^5^ Department of radiotherapy Fudan University Shanghai Cancer Center Shanghai China; ^6^ CAS Key Laboratory of Genomic and Precision Medicine, Collaborative Innovation Center of Genetics and Development, School of Future Technology, Beijing Institute of Genomics University of Chinese Academy of Sciences Beijing China; ^7^ Beijing Tiantan Hospital Capital Medical University Beijing China; ^8^ People's Hospital of Ningxia Hui Autonomous Region Ningxia Medical University Ningxia China; ^9^ Department of Pathology and Laboratory Medicine, Department of Surgery (Urology) BrownUniversity Warren Alpert Medical School and the Legorreta Cancer Center, Brown University Providence Rhode Island USA

**Keywords:** early noninvasive detection, *PCDHGB7* evaluation, urothelial carcinoma

## Abstract

Urothelial carcinomas (UCs) are the fourth most common male malignancies. However, currently implemented detection methods for UC are usually invasive and/or show passable sensitivity and specificity. An accurate, practical, and effective approach is urgently needed for UC clinical detection. Based on the observation that *PCDHGB7* was hypermethylated in UC, we developed a bisulfite‐free quantitative polymerase chain reaction (qPCR)‐based *PCDHGB7* evaluation to enable urine for UC noninvasive detection. A total of 887 urine samples from UC/benign diseases of the urinary system (BUD) patients between 2022 and 2023 were included. All collected samples were divided into training and validation sets in a 2:1 ratio based on the order of patient enrollment. Results showed that hypermethylated *PCDHGB7* exhibited excellent sensitivity of 87.3% (95% CI: 80.7%–92.3%) and specificity of 91.0% (95% CI: 84.8%–95.3%) in efficiently distinguishing UC from BUD patients in the validation set, which is highly consistent with its performance in the training set. Moreover, *PCDHGB7* hypermethylation showed promising potential in identifying sessile UC tumors or cases that might be missed in clinical detection and outperformed standard urine cytology in detecting bladder cancer (82.1% vs. 34.5%), ureter cancer (78.1% vs. 34.4%), and renal pelvis cancer (90.9% vs. 22.7%). Overall, bisulfite‐free qPCR‐based *PCDHGB7* evaluation in urine provided a noninvasive, easy‐to‐perform, and effective way for UC early detection.

**Translational Impact Statement**

This study introduced *PCDHGB7* evaluation, a qPCR‐based detection that enables urine for urothelial carcinomas (UC) early detection in a noninvasive and low‐cost way. It not only outperformed urine cytology, but also showed promising potential in the detection of problem‐awaiting UCs. The application of *PCDHGB7* evaluation is expected to lower the burden on patients and the healthcare system.

## 1 INTRODUCTION

Originating from the carcinogenesis of the urothelial epithelium of the urinary tract, urothelial carcinomas (UCs) are the fourth malignancies in males all over the world.^1^, ^2^ About 90% of UCs are urinary bladder cancer (UBC), and 10% are upper tract urothelial carcinoma (UTUC), including renal pelvic carcinoma and ureteral carcinoma.^3^ As the largest proportion of UC, approximately 75% of bladder cancers present with disease confined to the mucosa or submucosa, known as non‐muscle invasive bladder cancer (NMIBC), presenting a challenging management due to its high recurrence.^4^ Meticulous and stringent follow‐up checkups with repeat cystoscopy and urine cytology were recommended to timely intervene to prevent NMIBC upstaging. The remaining 25% is aggressive muscle‐invasive bladder cancer (MIBC) with high mortality.^5^ Cystoscopy combined with pathological biopsy is the gold standard for the diagnosis and follow‐up of UBC. However, the invasiveness, discomfort, and high‐cost natures of cystoscopy detection also decrease the patients' compliance.^6^ Furthermore, subtle and invisible neoplastic changes, particularly in carcinoma in situ (CIS) defined as a flat, high‐grade, and noninvasive UC, could be missed or misinterpreted as an inflammatory lesion by cystoscopy, which poses a potential threat of disease progression or recurrence.^5^, ^7^ In spite of the relatively low incidence, the insidious onset of UTUC, along with its poor prognosis, presents enormous challenges in clinical management.^8^, ^9^, ^10^ Neither cytology nor fluorescence in situ hybridization (FISH) shows high sensitivity for UTUC detection, while employing invasive diagnostic ureteroscopy (URS) plus biopsy before radical nephroureterectomy (RNU) would lead to an increased risk for intravesical recurrence.^11^ Thus, there is an urgent need to introduce a noninvasive, sensitive, and low‐cost way for UC detection.

Urine, which contains urothelial cells shed from urinary tracts directly and can be acquired noninvasively, emerges as an ideal sample type for UC detection.^12^ Urine cytology is the widely implemented alternative; however, the sensitivity of which is far from satisfactory in the clinical setting.^13^ Despite the Food and Drug Administration (FDA) having approved six urinary assays for clinical in vitro diagnostic use, these assays are unable to detect low‐grade malignancies and tend to give false positive results for benign inflammatory conditions.^14^, ^15^ With the discovery of epigenetic aberrations during the urothelium cancerization,^16^, ^17^ a number of DNA methylation‐based methods have been developed^18^, ^19^ but rarely have been adopted into clinical application owing to insufficient sensitivity/specificity and/or high expense.^20^

Previously, we revealed that *PCDHGB7* served as a universal cancer‐only marker (UCOM) was hypermethylated in multiple cancer types.^21^ In this study, we investigate whether methylated *PCDHGB7* could function for early detection of UC. We systematically evaluated the performance of hypermethylated *PCDHGB7* in the UC diagnoses and its potential applications in the clinic.

## 2 METHODS

### 2.1 Study design and participants

In this prospective and blinded study, all patients meeting all of the following inclusion criteria were recruited between March 2022 and August 2023 from Shanghai Changhai Hospital: (1) individuals of either gender, aged over 18 years old, and willing to provide 30–50 mL of fresh voided urine samples; (2) patients clinically diagnosed with benign diseases of the urinary system (BUD, consisting of urinary stones, cystitis glandularis, benign prostatic hypertrophy, and other benign diseases of the urinary system) or suspected of UC (including UTUC due to asymptomatic hematuria, upper urinary tract masses, hydronephrosis, pain over renal region, kidney failure, or other symptoms and UBC due to asymptomatic hematuria, bladder‐occupied lesions, urinary irritation symptoms, or other symptoms), without concurrent malignant tumors or any history of malignant tumors. Urine samples were collected before patients received any therapeutics, such as transurethral resection of bladder tumor (TURBT), cystectomy, systemic chemotherapy, immunotherapy, or intravesical infusion therapy. Urine samples were blinded when being transferred to laboratory personnel for methylation detection. During this step, specimens with one of the following criteria were excluded: (1) samples with failed quality control (DNA content of urine samples less than 100 ng); (2) failed assay (contamination of urine DNA caused by urinary tract infection or the insufficient methylation‐sensitive restriction endonuclease [MSRE] digestion). Two months after the last patient was enrolled, the sample information was unblinded. Patients meeting any of the following criteria were excluded: (1) unclear diagnosis or incomplete pathological information; (2) pathological diagnosis of malignant tumors other than UC, such as prostate cancer, renal carcinoma, and specific types of bladder cancer (including squamous cell carcinoma, adenocarcinoma, or small cell carcinoma, which do not originate from transitional cells in the inner lining of the bladder wall). Finally, the methylation results and clinical information of eligible patients were included in the analysis. This study was approved by the Ethics Committee of Shanghai Changhai Hospital (License Number: CHEC2022‐033). This study has been registered on ClinicalTrials.gov (ChiCTR2100052507).

### 2.2 Voided urine cytology

Urine cytology samples were collected from patients' spontaneous fresh voided urine and performed by three senior pathologists following The Paris System for Reporting Urinary Cytology published in 2022.^22^ Urinary cytology diagnostic categories were recorded as “intermediate to high risk” and “low risk” according to criteria in Table S3, using them for comparison with the diagnostic performance of *PCDHGB7* (comparing the sensitivity of “intermediate to high risk” in urine cytology with “positive” in *PCDHGB7* diagnosis and the sensitivity of “low risk” in urine cytology with “negative” in *PCDHGB7* evaluation).

### 2.3 DNA extraction

A total of 30–50 mL of early morning urine was collected with a urine collection tube (Epiprobe Biotech, K‐32). The urine sample was centrifuged at 1000*g* for 10 min and the pellet was retained for genomic DNA (gDNA) extraction according to the manufacturer's instructions of the Genomic DNA Extraction Kit (Epiprobe Biotech, A‐02). DNA extraction from frozen tissue samples was conducted by the QIAGEN DNA extraction kit (Qiagen, 51306 and 56404).

### 2.4 Bisulfite‐PCR pyrosequencing and DNA methylation evaluation

A total of 100 ng of gDNA was bisulfite‐converted using the EZ DNA Methylation‐Gold kit (ZYMO Research, D5006) following the manufacturer's instructions. Roughly 50 ng of recovered bisulfite‐treated DNA was used for subsequent PCR amplification as described previously.^21^ Amplifications were performed using the *Taq* 2× Master Mix kit (NEB, M0270L). The PCR program was set as 30 s at 98°C for pre‐denaturation, followed by 45‐cycle amplification (98°C for 10 s, 58°C for 30 s, and 72°C for 30 s), and a final extension at 72°C for 3 min. The amplified PCR products were confirmed by 2% agarose gel electrophoresis and pyrosequencing using PyroMark™ Q24 ID (Qiagen). The methylation level was calculated as the average methylation value of Cytosine‐phosphate‐Guanosine (CpG) sites contained.

### 2.5 Methylation sensitive restriction endonuclease‐qPCR and *PCDHGB7* methylation evaluation

*PCDHGB7* methylation was performed by dedicated laboratory investigators who were masked to the results of clinical diagnoses until DNA methylation detection was completed. *PCDHGB7* methylation in urine was quantified by MSRE‐qPCR using 100 ng gDNA digested with *Hha*I (NEB, R0139) and *Hpa*II (NEB, R0171) endonuclease. *GAPDH* gene, absent of any MSRE cutting sites, was designed for normalization (*GAPDH*_N_). A conservative hypomethylated region with cutting sites in *GAPDH* was designed as a quality control site (*GAPDH*_Q_) for the evaluation of MSRE digestion. MSRE‐qPCR primers/probes for targets were listed in Table S7. Subsequently, multiplex quantitative real‐time PCR with *PCDHGB7* and *GAPDH* primers/probes combination was performed on ABI 7500 Real‐Time PCR System (Applied Biosystems, Inc.) with a program of initiation at 95°C for 10 min and 45 cycles of amplification (94°C for 20 s and 60°C for 60 s). DNA methylation level was calculated with normalization to *GAPDH*, as described in the following formula: ΔCt_(*PCDHGB7* methylation level)_ = Ct__*PCDHGB7*_ – Ct__*GAPDH*N_. Each sample was run in three technical replicates.

### 2.6 Human methylation 450 K array analysis

The human methylation 450 K array data were obtained from The Cancer Genome Atlas (TCGA) and Gene Expression Omnibus (GEO) database. The absolute methylation level of *PCDHGB7* was calculated by the mean values of probes within the *PCDHGB7* promoter region (chr5:141417842‐141417900, GRCh38/hg38), and the final methylation value was calculated as ((*β* value + 0.5) × 100%). Detailed methylation values of all UC samples used from the TCGA and GEO databases were listed in Tables S1 and S2.

### 2.7 Limit of detection determination

gDNA from the bladder cancer cell line T24 was used as a positive control, and urothelial cell from the urine of a healthy volunteer (hereafter referred to as normal urothelial cell) was chosen as a negative control. Cancer cell DNA (DNA from T24) was gradient mixed with an amount of 0%, 0.2%, 0.5%, 1.0%, 5.0%, 10%, 20%, 30% and 100% of normal urothelial cell DNA. Bisulfite‐PCR pyrosequencing and MSRE‐qPCR were simultaneously conducted to determine the limit of detection (LOD).

### 2.8 Statistics

We divided the samples into training and validation sets in a 2:1 ratio based on the order of patient enrollment. Specifically, patients enrolled from March 2022 to March 2023 were assigned to the training set (*n* = 549), and those enrolled from April 2023 to August 2023 were assigned to the validation set (*n* = 275). Youden index, defined as ([sensitivity + specificity] – 1), was calculated to determine the optimal *PCDHGB7* methylation level threshold for distinguishing UC from BUD from the training set. The validation set was used to evaluate the diagnostic accuracy of urinary *PCDHGB7* methylation levels at the optimal cutoff (ΔCt = 3.01) for detecting UC. The metrics used for evaluation included sensitivity, specificity, positive predictive value (PPV), negative predictive value (NPV), and diagnostic accuracy. Receiver operating characteristic (ROC) curve was analyzed using the hybrid Wilson/Brown method. Differences between two groups were analyzed by two‐tailed unpaired Student's *t*‐test/nonparametric Mann–Whitney–Wilcoxon test, and one‐way analysis of variance (ANOVA) followed by Dunnett's multiple comparisons tests were performed when more than two groups were compared. All statistical analyses and data visualizations were carried out in R (3.6.0) with R packages and GraphPad Prism 9, and a *p* value less than 0.05 was considered significant (*p < 0.05; **p < 0.01; ***p < 0.001; and ****p < 0.0001).

## 3 RESULTS

### 3.1 Hypermethylated *PCDHGB7* exhibits applicational potential in UC diagnosis

**FIGURE 1:**
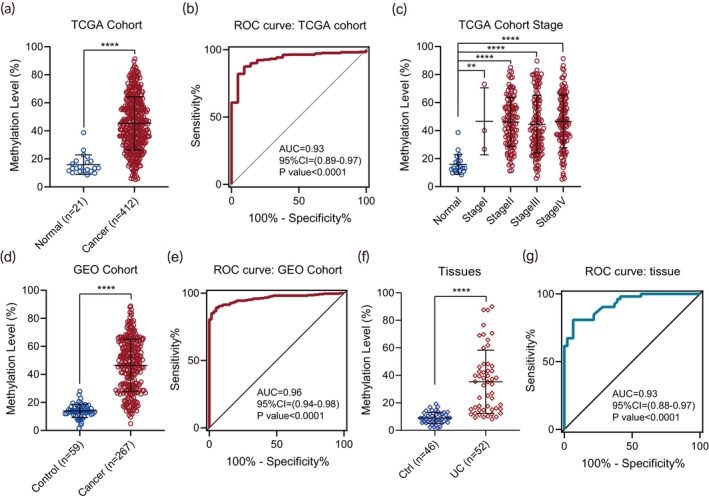
Hypermethylated *PCDHGB7* showed the potential of urothelial carcinoma (UC) detection. (a) *PCDHGB7* methylation in bladder urothelial carcinoma (BLCA) cohort samples from The Cancer Genome Atlas (TCGA) database. (b) The AUC value for distinguishing bladder cancer from normal in TCGA BLCA cohort. (c) Methylation level of *PCDHGB7* in TCGA BLCA cohort samples stratified by stage. (d) *PCDHGB7* hypermethylation was confirmed in UC samples from Gene Expression Omnibus (GEO) database. (e) Receiver operating characteristic (ROC) curve and the associated area under the curve (AUC) value of *PCDHGB7* in GEO data sets. (f) Methylation level of *PCDHGB7* was further validated in clinical UC samples by bisulfite‐PCR pyrosequencing. (g) The AUC values for distinguishing UC from para‐cancer tissues. Scatter dot plot shows the mean ± SD. *p* values were calculated using the two‐tailed nonparametric Mann–Whitney test. **, *p* < 0.01; ****, *p* < 0.0001.

DNA methylation aberration of specific genes occurs during the cancerous initiation and progression stages,^23^ making it a favorable target for cancer screening. Our previous work identified that *PCDHGB7* as a UCOM marker was significantly hypermethylated in multiple types of cancers. Given the unsatisfactory sensitivity of urine cytology and the invasive nature of cystoscopy/URS plus pathological biopsy, we aimed to investigate the applicability of *PCDHGB7* in UC detection. Methylation 450 K array data from two different public data sets indicated that *PCDHGB7* was hypermethylated in UC (Figure 1a,d; Tables S1 and S2), when compared to normal controls. ROC curve analysis demonstrated that *PCDHGB7* methylation levels exhibit excellent diagnostic performance in both data sets, with area under the curve (AUC) values of 0.93 (95% CI: 0.89–0.97) (Figure 1b) and 0.96 (95% CI: 0.94–0.98) (Figure 1e), respectively. Not only that, *PCDHGB7* has already been hypermethylated in the early stage of UC (Figure 1c). Next, we further collected 46 para‐cancer (normal urothelial tissues adjacent to the UC) and 52 UC tissues from the clinic to analyze the DNA methylation status of *PCDHGB7* by bisulfite‐PCR pyrosequencing. It showed that *PCDHGB7* was significantly hypermethylated in UC compared to the para‐cancer tissues (Figure 1f). When applying *PCDHGB7* to discriminate UC from para‐cancer tissues, the AUC was 0.93 (95% CI: 0.88–0.97) (Figure 1g). Collectively, these results supported the applicational potential of hypermethylated *PCDHGB7* in UC diagnosis.

### 3.2 Bisulfite‐free *PCDHGB7* methylation detection with 0.2% resolution

**FIGURE 2:**
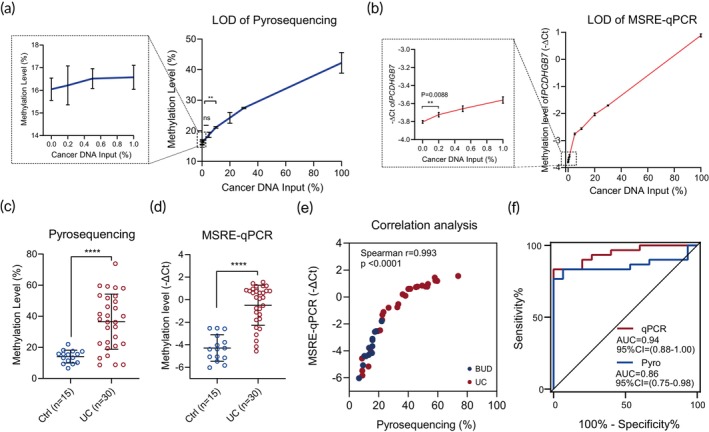
Development of high‐resolution methylation detection. (a, b) Limit of detection (LOD) determination of *PCDHGB7* by bisulfite‐PCR pyrosequencing (a) and methylation‐sensitive restriction endonuclease (MSRE)‐qPCR (b). The higher value of calculated –ΔCt corresponded to a higher methylation status. (c, d) *PCDHGB7* methylation level in urine samples of urothelial carcinoma (UC) and benign diseases of the urinary system (BUD) patients detected by bisulfite‐PCR pyrosequencing (c) and MSRE‐qPCR (d). (e) Correlation analysis in urine samples detected by bisulfite‐PCR pyrosequencing and MSRE‐qPCR. Spearman's rank correlation coefficient and *p* value were calculated using GraphPad Prism. (f) The receiver operating characteristic (ROC) curve of pyrosequencing and MSRE‐qPCR in detecting *PCDHGB7* methylation level. The mean ± SD values were plotted. *p* values were calculated using the two‐tailed unpaired Student's *t*‐test. **p < 0.01; ****p < 0.0001; ns, not significant.

Diagnosing cancer on the basis of cancer‐derived DNA is challenging due to the extremely limited amount of cancerous DNA.^24^ Additionally, methylation conducted by bisulfite‐PCR pyrosequencing is time‐consuming and unstable to facilitate in a clinical setting.^25^ Herein, we optimized the reaction system of the restriction enzyme‐based multiplex qPCR to keep the consistency in quantification cycle values (Ct), linearity, sensitivity, and efficiency of assay performance of the singleplex and multiplex reactions (Figure S1; Table S4), which makes it save setup time, reduce cost, and minimize samples' consumed amount. To ensure the sensitivity of the methodology, LOD was detected. It turned out that as little as 0.2% of cancerous DNA component could be detected by restriction enzyme‐based bisulfite‐free qPCR stably (Figure 2b). Bisulfite‐PCR pyrosequencing of *PCDHGB7*, by contrast, was much less sensitive, which tends to be undetectable when cancer DNA was less than 5% (Figure 2a). Subsequently, we detected the *PCDHGB7* methylation level in urine samples from UC patients and BUD controls by MSRE‐qPCR and bisulfite pyrosequencing, respectively. It turned out that *PCDHGB7* was hypermethylated in urinary sediment of cancer cases (Figure 2c,d). MSRE‐qPCR‐based results were highly correlated with the methylation levels detected by pyrosequencing (Figure 2e). In addition, the AUC of MSRE‐qPCR (0.94, 95% CI: 0.88–1.00) was markedly higher than that of bisulfite pyrosequencing (0.86, 95% CI: 0.75–0.98) (Figure 2f). These results indicated that it is superior to trace the microscale cancerous DNA by bisulfite‐free *PCDHGB7* methylation detection in urine.

### 3.3 *PCDHGB7* hypermethylation with high efficiency in UC detection

**FIGURE 3:**
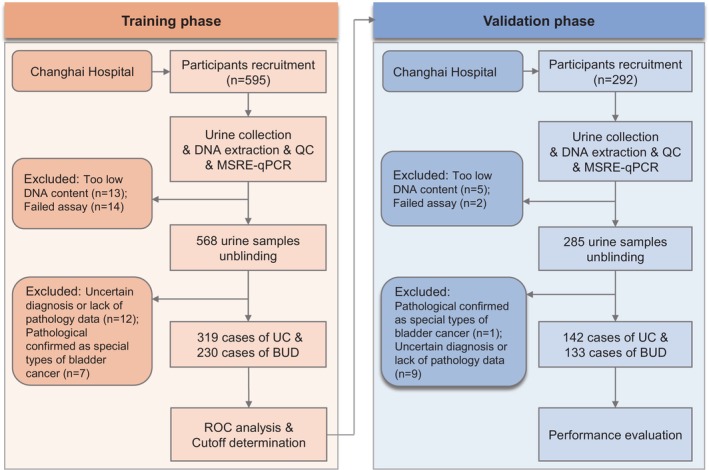
Workflow of the study design. BUD, benign diseases of the urinary system; MSRE‐qPCR, methylation‐sensitive restriction enzyme qPCR; QC, quality control; ROC, receiver operating characteristic; UC, urothelial carcinoma.

**FIGURE 4:**
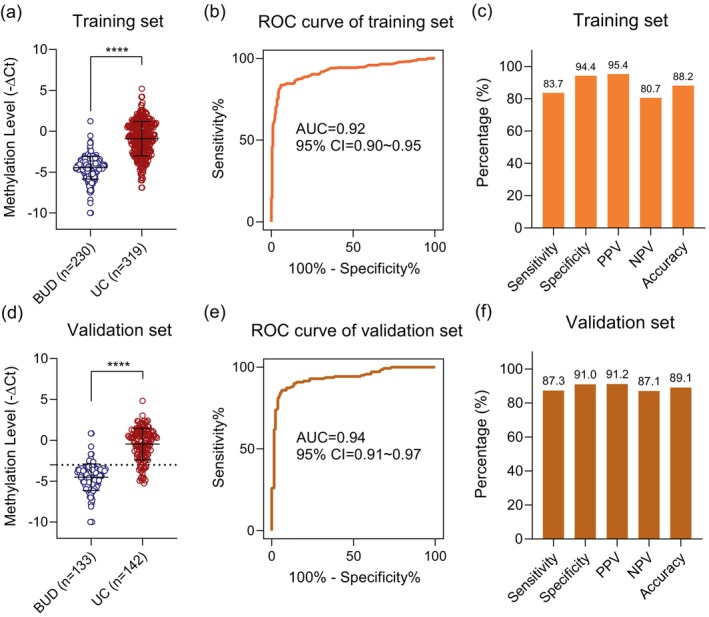
Performance of *PCDHGB7* evaluation in urine for urothelial carcinoma (UC) detection. (a) Methylation level of *PCDHGB7* represented by –ΔCt was detected by methylation‐sensitive restriction enzyme (MSRE)‐qPCR in the training set. The higher value of calculated –ΔCt corresponded to a higher methylation status. (b) Receiver operating characteristic (ROC) curve analysis of *PCDHGB7* in the training set. (c) Performance of *PCDHGB7* in the training set. (d) *PCDHGB7* methylation level detected in the validation set. (e) ROC curve analysis of *PCDHGB7* in the validation set. (f) The sensitivity, specificity, accuracy, positive predictive value (PPV), and negative predictive value (NPV) of *PCDHGB7* evaluation in the validation set. Scatter dot plot shows the mean ± SD. *p* values were calculated using the two‐tailed nonparametric Mann–Whitney test. ****p < 0.0001. BUD, benign diseases of the urinary system.

To investigate the practicability of *PCDHGB7* in UC noninvasive detection, we collected urine for the establishment of *PCDHGB7* evaluation. The study design and implementation are summarized in Figure 3. In the training phase, 549 qualified urine samples, including 319 UC and 230 BUD, were enrolled from Shanghai Changhai Hospital (Table S5). Methylation detection showed that *PCDHGB7* was significantly hypermethylated in UC (Figure 4a). Additionally, the ROC curve exhibited a high AUC of 0.92 (95% CI: 0.90–0.95), which could effectively distinguish UC from BUD patients with a sensitivity of 83.7% (95% CI: 79.2%–87.6%) and a specificity of 94.4% (95% CI: 90.5%–97.0%) (Figure 4a–c).

To further verify the performance of *PCDHGB7*, a total of 275 cases were intended for validation. Detailed clinical characteristics of participants are shown in Table S5. Consistently, prominent *PCDHGB7* hypermethylation was also the case in UC (Figure 4d). *PCDHGB7* showed excellent ability to distinguish UC from BUD (AUC = 0.94; 95% CI: 0.91–0.97) (Figure 4e). Urine detection presented with satisfactory performance with a sensitivity of 87.3% (95% CI: 80.7%–92.3%), a specificity of 91.0% (95% CI: 84.8%–95.3%), a PPV of 91.2% (95% CI: 85.1%–95.4%), an NPV of 87.1% (95% CI: 80.3%–92.1%), and an overall accuracy rate of 89.1% (95% CI: 84.8%–92.5%) in the validation set (Figure 4f). The high consistency between the training and validation sets suggested *PCDHGB7* can efficiently distinguish BUD from UC samples in a noninvasive manner.

### 3.4 *PCDHGB7* as a promising marker in clinical problem‐awaiting scenarios

**FIGURE 5:**
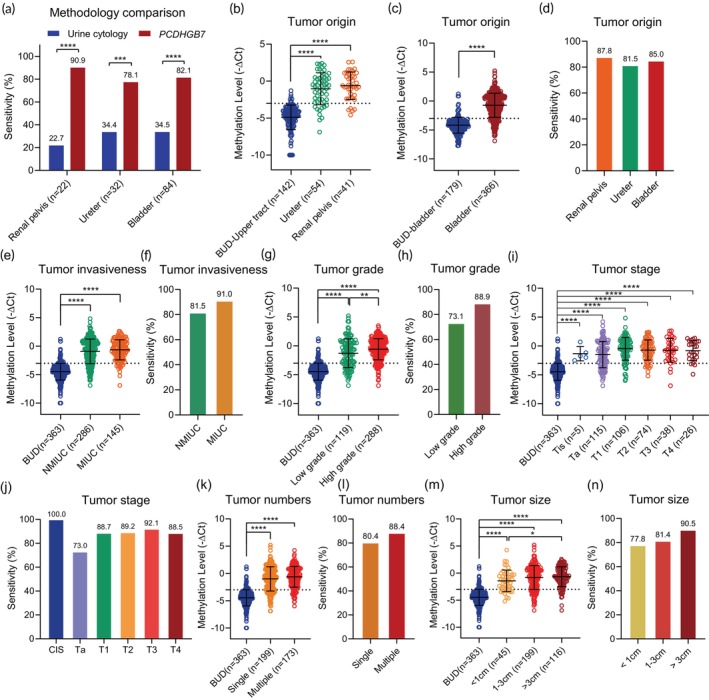
Performances of *PCDHGB7* evaluation in clinical problem‐awaiting scenarios. (a) Methodology comparison between urine cytology and *PCDHGB7* methylation with regard to sensitivity in upper urinary tract (renal pelvis and ureters) and bladder cancer. (b–d) Methylation level of *PCDHGB7* and sensitivity were assessed in renal pelvis cancer, ureter cancer, and bladder cancer. (e, f) *PCDHGB7* methylation and sensitivity in non‐muscle invasive urothelial carcinomas (NMIUC) and muscle‐invasive urothelial carcinoma (MIUC). (g, h) Methylation level of *PCDHGB7* as well as sensitivity were analyzed following tumor grade. (i, j) *PCDHGB7* methylation and sensitivity in different tumor stages. (k, l) Methylation level of *PCDHGB7* and sensitivity in single‐focal and multi‐focal UCs. (m, n) *PCDHGB7* methylation as well as sensitivity in different max diameters of urothelial carcinomas (UCs). Scatter dot plot shows the mean ± SD. Differences between two groups were analyzed by two‐tailed unpaired Student's *t*‐test, and one‐way ANOVA followed by Dunnett's multiple comparisons tests were performed when more than two groups were compared. *p < 0.05; **p < 0.01; ***p < 0.001; ****p < 0.0001; ns, not significant. CIS, carcinoma in situ; BUD, benign diseases of the urinary system.

We firstly compared the performance of *PCDHGB7* evaluation and urine cytology in a sub‐cohort containing 138 UC patients. Results showed that *PCDHGB7* evaluation outperformed urine cytology in bladder cancer (82.1% vs. 34.5%), ureter cancer (78.1% vs. 34.4%), and renal pelvis cancer (90.9% vs. 22.7%) (Figure 5a), indicating this methylation‐dependent detection had higher efficiency.

Although rare, UTUC showed a more aggressive phenotype and lower survival rate than bladder cancer, and therefore, early detection of UTUC is in dire need. For this purpose, UC samples were reanalyzed according to the tumor origin; both renal pelvis cancer and ureter cancer showed significantly higher methylation levels of *PCDHGB7* with a sensitivity of 87.8% (95% CI: 73.8%–95.9%) and 81.5% (95% CI: 68.6%–90.8%), respectively (Figure 5b–d and Table S6), demonstrating that *PCDHGB7* evaluation could provide an efficient detection method for UTUC management in a noninvasive way. Accounting for approximately 80% of UC,^5^, ^26^ non‐muscle invasive urothelial carcinomas (NMIUC) are prone to recur and be missed by urine cytology detection. Herein, NMIUC and MIUC accompanied by significantly hypermethylated *PCDHGB7* showed a sensitivity of 81.5% (95% CI: 76.5%–85.8%) for NMIUC and a sensitivity of 91.0% (95% CI: 85.2%–95.1%) for MIUC (Figure 5e,f and Table S6), indicating that *PCDHGB7* could be a good candidate for NMIUC detection. As anticipated, *PCDHGB7* evaluation exhibited outstanding performance in detecting high‐grade tumors with a superior sensitivity of 88.9% (95% CI: 84.7%–92.3%). Besides, low‐grade tumors with a similar appearance and organization to normal cells were detected with a good sensitivity of 73.1% (95% CI: 64.2%–80.8%) (Figure 5g,h and Table S6).

As for CIS with flat lesion and particularly high rates of progression, despite the relatively limited number of subjects, all samples were accurately diagnosed with a sensitivity of 100.0% (95% CI: 47.8%–100.0%). More detailed, the *PCDHGB7* evaluation showed a sensitivity of 73.0% (95% CI: 64.0%–80.9%) in Ta‐stage, 88.7% (95% CI: 81.1%–94.0%) in T1‐stage, 89.2% (95% CI: 79.8%–95.2%) in T2‐stage, 92.1% (95% CI: 78.6%–98.3%) in T3‐stage, and 88.5% (95% CI: 69.9%–97.6%) in T4‐stage (Figure 5i,j and Table S6), which pointed out that *PCDHGB7* evaluation could be applied to the detection of tumors at different stages, even for the early stage of Ta and T1.

Roughly one‐third of all bladder cancers occur as a multi‐focal disease, which increases the difficulty in management.^27^ Here, the sensitivity of *PCDHGB7* evaluation was 80.4% (95% CI: 74.2%–85.7%) for single‐focal UC and 88.4% (95% CI: 82.7%–92.8%) for multi‐focal UC (Figure 5k,l and Table S6). Moreover, detection sensitivity varied with tumor size, for which the maximum diameter (*D*_max_) above 3 cm showed the highest methylation level and presented with a sensitivity of 90.5% (95% CI: 83.7%–95.2%). While for tumors with a diameter below 1 cm that might be missed by ultrasound or even cystoscopy in clinical diagnosis, *PCDHGB7* evaluation also showed a high sensitivity of 77.8% (95% CI: 62.9%–88.8%) (Figure 5m,n and Table S6), revealing the superiority of *PCDHGB7* evaluation even in the early stage of tumors. Collectively, these results suggested the promising potential of *PCDHGB7* hypermethylation, especially for sessile, low‐grade, and small UC tumors in clinical detection.

## 4 DISCUSSION

The current “gold standard” for UC diagnosis and follow‐up involves cystoscopy and urine cytology,^5^, ^28^ which have been widely blamed for their invasiveness and low sensitivity, respectively. Although the FDA has approved six urine assays, the problem of identifying low‐grade tumors persists due to the inclusion of large proportions of high‐grade tumors inflating the sensitivity and specificity.^14^ Herein, *PCDHGB7* evaluation showed superior sensitivity of 88.9% in high‐grade UC and passable sensitivity of 73.1% in low‐grade UC. In addition, many biomarkers are associated with high false positivity rates because they are susceptible to bladder mucosal inflammation, leading to overdiagnosis.^15^ By contrast, *PCDHGB7* methylation detection showed a high specificity of 91.0% and a low false positivity rate of 9.0%. Using a panel of multiple genetic and/or epigenetic markers^18^, ^19^, ^29^, ^30^ overcomes the former issue and yields acceptable performance; however, most of these assays need a complicated model construction process and somewhat undermine the principle that a screening test should be simple, accessible, and reasonably cost‐effective.^15^ Here, we proposed *PCDHGB7* hypermethylation to enable urine for UC detection in a noninvasive, easy‐to‐perform, and effective way. Based on bisulfite‐free qPCR platform with a rapid 3 hour turnaround time and the use of existing laboratory infrastructure, *PCDHGB7* evaluation exhibited high sensitivity of 87.3% and specificity of 91.0% in distinguishing UC from BUD patients. Additionally, *PCDHGB7* evaluation has the potential to detect flat (such as CIS)^31^ and low‐grade tumors^32^ that were in the early stage of UC progression and might be overlooked by urine cytology.

Flexible URS combined with biopsy may contribute to the diagnosis of UTUC and the decision‐making process between RNU and kidney‐sparing therapy. However, it would lead to an increased risk for intravesical recurrence if URS was performed before RNU.^33^ *PCDHGB7* evaluation provides chances for early detection of UTUC by capturing the tiny changes in DNA methylation of exfoliated cells from urine. Especially in situations where radiography fails to provide a definitive diagnosis, *PCDHGB7* may assist urological clinicians in disease assessment, potentially avoiding the discomfort of a URS and the risk of postoperative intravesical recurrence for patients.

Despite the merits mentioned above, a limitation of this study is that the analyzed samples were small and information of enrolled patients was incomplete. The practicability of *PCDHGB7* needs to be further validated in a larger multicenter prospective cohort. Furthermore, a long‐term follow‐up observation is needed to provide more clinical values of *PCDHGB7* methylation.

Taken together, our study uncovered that *PCDHGB7* evaluation conducted by bisulfite‐free qPCR in urine emerges as a powerful approach for UC early noninvasive detection. Expectantly, its implementation is expected to improve UC management.

## 5 CONCLUSION

Our study proposed *PCDHGB7* evaluation, a noninvasive and easy‐to‐perform approach to enable UC detection in urine samples with high efficiency and convenience. It provided an alternative for problem‐awaiting UCs with unmet needs clinically, and its application is expected to lower the burden on patients and the healthcare system.

## AUTHOR CONTRIBUTIONS

**Zhicong Yang:** Software; data curation; writing – original draft; writing – review and editing; validation; methodology; formal analysis. **Qing Chen:** Writing – review and editing; data curation; visualization. **Shihua Dong:** Writing – original draft; investigation; supervision; methodology. **Peng Xu:** Writing – review and editing; data curation. **Zhanrui Mao:** Data curation. **Yaping Dong:** Methodology. **Wei Li:** Methodology. **Wenxuan Li:** Software. **Yang Han:** Data curation. **Lihe Dai:** Data curation. **Gehong Dong:** Data curation. **Yong Zhang:** Data curation. **Yinshan Li:** Resources. **Liang Cheng:** Supervision; conceptualization. **Weimin Ci:** Conceptualization; supervision. **Wenqiang Yu:** Conceptualization; supervision; project administration; writing – review and editing; resources. **Chuanliang Xu:** Conceptualization; supervision; funding acquisition; project administration; writing – review and editing.

## CONFLICT OF INTEREST STATEMENT

Shihua Dong used to be the R & D director of Epiprobe. Peng Xu used to be an employee of Epiprobe. Wenqiang Yu serves on the scientific advisory boards of Epiprobe. All other authors declare no competing interests.

## Supporting information


**Table S1.** Detailed methylation level of *PCDHGB7* in TCGA data sets.
**Table S2**. Detailed methylation level of *PCDHGB7* in GEO data sets.
**Table S3**. Criteria used for interpretation of urine cytology.
**Table S4**. PCR efficiency and coefficient of correlation for singleplex or multiplex MSRE‐qPCR with human cell line T24 DNA.
**Table S5**. Clinical characteristics of the training and validation set.
**Table S6**. The performance of *PCDHGB7* in diverse UC subgroups.
**Table S7**. MSRE‐qPCR primers/probes for targets.

**Figure S1.** Ct values from MSRE‐qPCR with a six‐point standard curve of human cell line T24 DNA. VIC‐*PCDHGB7* primer/probe set (A) and CY5‐*GAPDH* primer/probe set (B) were used for singleplex or multiplex reactions.

## Data Availability

The data that support the findings of this study are available from the corresponding author upon reasonable request.
