# Supplementary material for: Bisulfite‐free PCDHGB7 methylation in urine enables early noninvasive detection of urothelial carcinoma
Source: Bioeng Transl Med. 2025 Feb 26;10(4):e70004. doi: 10.1002/btm2.70004 (PMC12284432; doi:10.1002/btm2.70004)
Supplement: Supplementary file 2 — Figure S1. Ct values from MSRE‐qPCR with a six‐point standard curve of human cell line T24 DNA. VIC‐PCDHGB7 primer/probe set (A) and CY5‐GAPDH primer/probe set (B) were used for singleplex or multiplex reactions. [file BTM2-10-e70004-s002.docx]

**Bisulfite-free *PCDHGB7* methylation in urine enables early noninvasive detection of urothelial carcinoma**

Zhicong Yang^1†^, Qing Chen^2†^, Shihua Dong^1,3†^, Peng Xu^1,3†^, Zhanrui Mao^1^, Yaping Dong^4^, Wei Li^5^, Wenxuan Li^1^, Yang Han^6^, Lihe Dai^2^, Gehong Dong^7^, Yong Zhang^7^, Yinshan Li^8^, Liang Cheng^9*^, Weimin Ci^6*^, Wenqiang Yu^1*^, and Chuanliang Xu^4*^


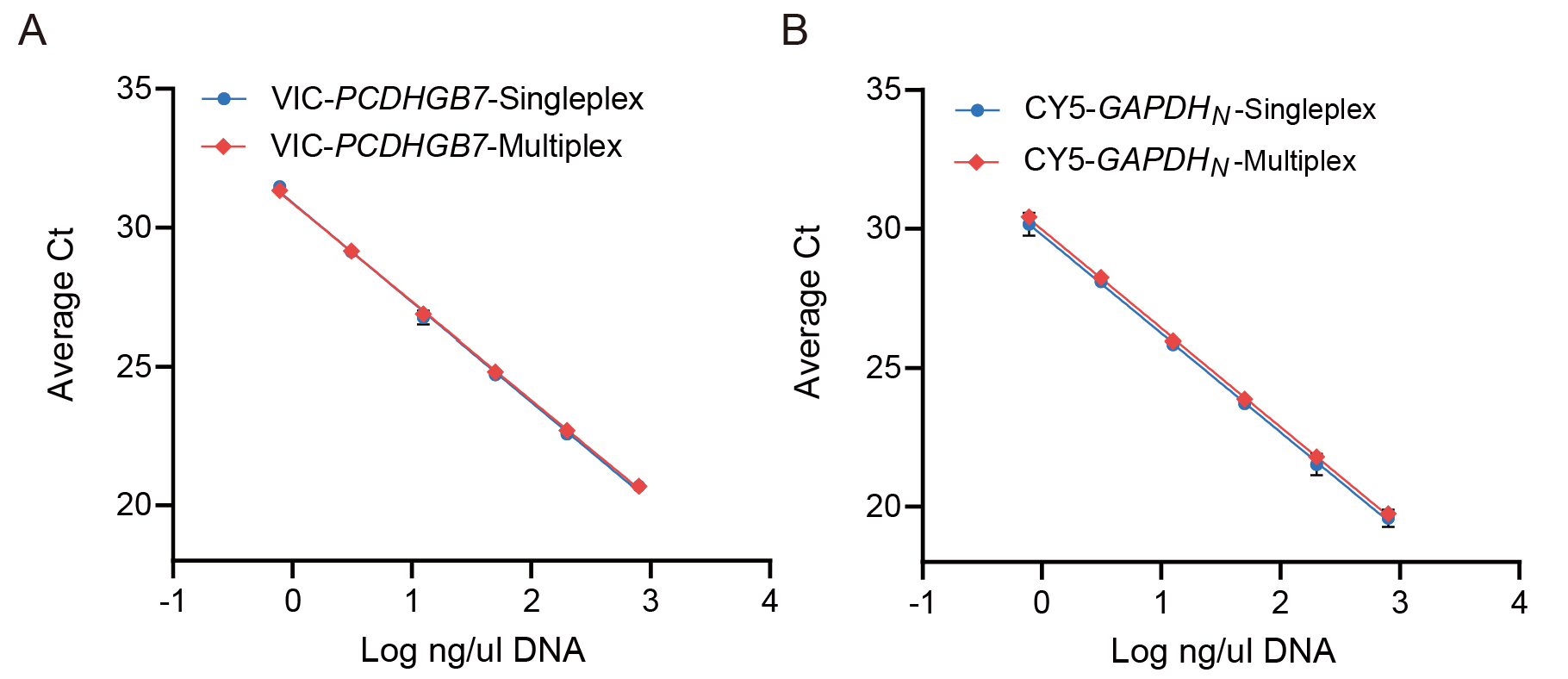


**Figure S1.** Ct values from MSRE-qPCR with a six-point standard curve of human cell line T24 DNA. VIC-*PCDHGB7* primer/probe set (A) and CY5-*GAPDH* primer/probe set (B) were used for singleplex or multiplex reactions.
